# The Chameleon Behavior of Sarcoidosis

**DOI:** 10.3390/jcm10132780

**Published:** 2021-06-24

**Authors:** Claudio Tana, Cosima Schiavone

**Affiliations:** 1 COVID-19 Medicine Unit and Geriatrics Clinic, S.S. Annunziata Hospital of Chieti, 66100 Chieti, Italy; 2 Unit of Ultrasound in Internal Medicine, Department of Medicine and Science of Aging, “G. d’Annunzio” University of Chieti, 66100 Chieti, Italy; cschiavone@unich.it

Sarcoidosis is a multisystem disease that raises several diagnostic difficulties in routine clinical practice due to its multisystemic involvement and the presence of nonspecific clinical pictures, except in some isolated cases [1]. The granulomatous involvement from sarcoidosis is the pathological finding that typically differentiates sarcoidosis from other disorders, but often a biopsy is necessary, a diagnostic procedure that is at potential risk of serious complications [2]. Granulomas are typically non-caseating and non-necrotizing, unlike other pathologies (e.g., tuberculosis), though sometimes non-caseating granulomas are also found in histopathological analysis of other disorders, making this differentiation even more complicated [3]. Lungs are the sites most often affected, and the restrictive impairment of respiratory function is frequently observed in an advanced phase of the disease [4,5,6]. Sarcoidosis can be misdiagnosed with a plethora of other disorders such as tuberculosis, chronic obstructive pulmonary disease (COPD), or less common and atypical diseases such as lymphangitic carcinomatosis, tumors, lymphoma and several others [7,8]. In the recent pandemic from the novel coronavirus disease from SARS-CoV-2, the differential diagnosis is even more difficult since these two disorders can coexist and give similar radiological findings [9,10,11]. Recent innovation in diagnostic techniques, such as computed tomography and magnetic resonance imaging and the introduction of artificial intelligence-based software that can help to trace non-invasive patterns of distinction between sarcoidosis and other disorders at imaging, is useful in the diagnostic workup and management of this disease [12]. The most recent novelties in the diagnostic and prognostic evaluation of pulmonary sarcoidosis, and an exhaustive presentation of clinical features, pathogenesis and therapy of sarcoidosis, are herein presented from some authors [13]. Moreover, Yamaguchi et al. report an interesting overview on the potential etiology-based diagnosis of sarcoidosis, based on histopathological demonstration of potential microbial antigens, giving new perspectives in the diagnosis and also for the antimicrobial intervention against sarcoidosis [14].

This issue is now open for submission, research articles and reviews are welcome for consideration, and the proposal of original articles and systematic review and metanalysis is particularly encouraged. Since sarcoidosis can show chameleon-like behavior, which could deceive even the most experienced clinicians [15,16], it can be helpful to report the most recent advances in the diagnosis and management of sarcoidosis, which may be of support to clinicians in their daily clinical practice.

## Data Availability

Not applicable.
